# Music as Fluidum: A Rheological Approach to the Materiality of Sound as Movement Through Time

**DOI:** 10.3390/bs15081118

**Published:** 2025-08-18

**Authors:** Mark Reybrouck

**Affiliations:** 1 Musicology Research Group, Faculty of Arts, KU Leuven-University of Leuven, 3000 Leuven, Belgium; mark.reybrouck@kuleuven.be; 2 Institute for Psychoacoustics and Electronic Music (IPEM), Department of Art History, Musicology and Theatre Studies, Faculty of Arts and Philosophy, 9000 Ghent, Belgium

**Keywords:** music and motion, fluidum, rheology, Deborah number, phoronomy, ideomotor simulation, organic form

## Abstract

Music is an elusive phenomenon with sounds that disappear while sounding. This challenges the description of the music and its processing by the listener or performer. A possible answer to this problem lies in the definition of music as flowing sound energy that continuously modifies its substance and shape. Such an approach adheres to the materiality of sound and allows for a description of music in rheological terms. We therefore take as a starting point the analogy of music as a virtual, motional object that follows a trajectory through time, revolving around three major issues: (i) the relation between sound and motion, (ii) the description of motion or movement over time, and (iii) the embodied and enactive character of musical engagement. The paper relies mainly on historical sources—most notably the work of Alexander Truslit on motion perception and Ernst Kurth on energetics—and connects them to modern paradigms of embodied and enactive cognition as applied to music.

## 1. Introduction

Music is the most elusive of all arts. Its intangible and ephemeral character—with sounds that disappear as they sound—challenges both the description of the music and its processing by the listener. In this paper, we highlight both approaches by stressing the definition of music as a temporal and sounding art. This means that we ground our discussion in the concept of “sounding moving forms,” paraphrasing somewhat Hanslick’s obscure and somewhat untranslatable phrase (*tönend bewegte Formen*) (Hanslick, 1854/1966). Stated in operational terms, this means that we conceive of music as flowing sound energy that continuously modifies its substance and shape. It is an approach that evokes an analogy to the science of *rheology*, which is the field of study that describes and assesses the flow and deformation of matter, primarily in a fluid state (see below). Taking flow (along with resistance, etc.) as the platform for an argument for how music flows and how listeners perceive this flow, however, raises questions about the essence of a musical flow (both empirically and phenomenologically). A first and obvious answer is to conceive of music in terms of a *fluidum*, which can be defined in the broadest sense as a substance (liquid or gas) whose particles can move about freely and that flows, deforms, and changes shape when subject to a force. The term aligns with the definition of a fluid in physics. There is, however, a distinction to be made between particles that can move freely in a fluidum and the free movements of sounds that are heard as tones (Scruton, 1999, p. 16). Sounds, in the latter case, can be seen as “events” with a temporal course and meaning, which can be very short or more extended over time. There is, as such, a distinction between the way listeners represent this flow: either as low-resolution global temporal spans (in the range of seconds or longer) or as high-resolution representations that operate at the level of the individual tones or even below that level (in the range of milliseconds). This is an interesting approach that refers to the *dynamics of representation*, which means that there is a multiplicity of representations, which range from “broad-band” overviews that represent longer stretches of temporal unfolding to “frame by frame” or “moment-to-moment” sequential scanning of the sounding particulars that make up the idiosyncrasies of the sonorous unfolding (Godøy, 1997). There is, as such, a continuum over step by step processing to synoptic overview.

Defining music in rheological terms, furthermore, raises several questions: What is the substance of music? Is it a solid, a liquid, or something between? Is it real or virtual? Can we assign some materiality to the sounds, and how do we engage with that matter in terms of a fluid that continually changes its momentary states? Terms like morphodynamics and fluid mechanics are likely to emerge in this regard, not only for the description of the actual sonorous unfolding but also by providing descriptive and assessment tools for mapping the wealth of listeners’ ways of musical sense-making. The terms, however, need some clarification both with respect to their meaning and their relation to music. In what follows, we start with the basic claim that music is *movement through time*. We then delve into the rheological and phoronomic properties of things in motion, first in general and then applied to music, to end with a double description of music as a continuous function of time and the consumption of time by the listener, which together make up the dynamics of listening.

### 1.1. Back to Basics: Sound Is More than Sound

A distinction must be made between intuitive descriptions that rely on “descriptive metaphors” for music, such as the river analogy, music as an organism, music as a virtual, motional object, etc., and more objective, “explanatory approaches” that rely on measurement and validation. The latter are the subject of much contemporary empirical research; the former are found more readily in older contributions, which have fallen somewhat into disrepute because of their lack of methodological sophistication. This lack of rigor of so-called “older” scholars, however, is often compensated by their depth of insight and breadth of view. This means that even if they may be largely speculative and subjective at times, their insights might be highly relevant to contemporary research (Repp, 1992b). In what follows, we therefore delve deeply into some seminal contributions that have often been quoted rather than being read. Examples are Truslit on music and motion (Truslit, 1938), Kant on phoronomy (Kant, 1786/1900), and Bingham on rheology (Bingham, 1922, 1944).

Generalizing a little, these early writings have emphasized three major claims: (i) the relation between sound and motion, (ii) the description of motion or movement over time, and (iii) the embodied and enactive character of musical engagement. Together, they can be used to describe the actual flow of the sounding music and the ongoing music processing by the listener. What matters, in this approach, is the *music as heard* or *listened to*, with a major focus on the musical experience rather than conceiving of the music in an abstract and detached way without direct connection to the actual articulation of the sounds (Reybrouck, 2014, 2019; Evans, 1990). A critical stance in this regard has been advocated by Small in his concept of *musicking* (Small, 1998), who tries to avoid the trap of *reification* of the music—conceiving of “works of music”—by stressing the activity we call music and whose meanings should be grasped in time as the music flies rather than being fixed on paper. It is an approach that goes against the long-celebrated and prevailing conception of German musicologists who stated that the subject matter of music is made up, primarily, of significant works of music that have outlived the culture of their age (Dahlhaus, 1982). Such an emphasis on the concept of work as a kind of “thingness” values the created art object rather than the action of art, the act of creating and that of perceiving and responding. Musicking, on the contrary, is more ambitious in its scope, or, as Small has put it: “To music is to take part, in any capacity, in a musical performance, whether by performing, by listening, by rehearsing or practicing, by providing material for performance (what is called composing), or by dancing” (Small, 1998, p. 9).

Taken together, these “activity-related” approaches align with the *phenomenological approach* to music listening, advocated since the 1960s (see, e.g., Batstone, 1969; Clifton, 1975, 1983; Ferrara, 1984; Green, 1982; Greer, 1984; D. Lewin, 1986; Schütz, 1951, 1971, 1976; Skarda, 1979; F. Smith, 1976). It is only recently, however, that the study of music based on phenomenological concepts and methods has received more prominence (Ihde, 2007). A primary focus, in this regard, is the emphasis on the human experience of *music as a heard phenomenon* (Lochhead, 1980, 1986) in an attempt to gain a more comprehensive understanding of the human mind by also considering consciousness and subjectivity. Such an approach goes beyond mere cognition by also encompassing emotion, affect, and motivation as factors that influence the way we think, perceive, act, or feel (LeDoux, 1996, 2002, p. 24). Subjectivity, in that view, cannot be understood without relation to unconscious psychological and bodily structures and processes, which means that it extends throughout the body with loops through the material, social, and cultural environments in which the body is embedded (Thompson, 2007, pp. 12 and 16–17).

The phenomenological approach, however, has many guises. In its original Husserlian conception, it adopts a *first-person approach* towards the world, which can be either “thematic” or “unthematic.” The latter is what Husserl termed the natural attitude as the kind of unreflective and straightforward immersion in a world that exists more or less independently of us; the former steps back from this natural attitude by investigating the actual experience. It is a critical and non-dogmatic attitude with attention being actively directed to the world strictly as we experience it. This means that we should be attentive to the relation between our subjectivity and the appearance or disclosure of the world, as exemplified in Husserl’s famous maxim “Back to the things themselves” (Husserl, 1950, 1970).

The phenomenological approach, therefore, is grounded in the way we *experience* things to provide a sensitive account of the various structures of our experience (Gallagher & Zahavi, 2008, p. 6). Adopting such an attitude amounts to being interested in the way things are experienced as relational correlates to our subjectivity. It entails a more flexible and trainable mental skill of turning our inattentive immersion of the natural attitude into a more deliberate attention to how something appears to us or is given to our experience (Thompson, 2007, pp. 18–20; see also Steinbock, 2004; Ihde, 2007; and Small, 1998 for music applications).

It is not difficult to translate this phenomenological stance to the realm of music and to conceive of music not merely in terms of sounds but of sounds that trigger our deliberate attention. Sounds, then, are more than sounds: they are sounds that may be able to capture and direct our attention. As such, they can obtain some kind of saliency that sets them apart from mere acoustic stimuli. Critical in such a directional and attentional stance, moreover, is the tension between the above-mentioned moment to moment “process” of listening in real time and the grasping of the broader “overview,” also called outside time processing, thus combining the perspectives of presence and distance, which together constitute the experience of time (Reybrouck, 2019). There is, as such, an interplay between musical detail, overview, and direction, as advocated already in the concept of *Fernhören* (remote listening), used formerly by Heinrich Schenker and Wilhelm Furtwängler. A large-scale musical work, therefore, cannot only be seen as an object; it is also a process of constant re-shaping and change (*Werden =* becoming, rather than *Sein* = being) through the workings of perception, memory, and expectation (Guldbrandsen, 2015, 2020).

Every interaction with music, therefore, implies a consciousness of time that is focused on the present, past, and future. Projecting memory contents into the future and anticipating forms of progress make listening an active process that translates the music into patterns of *growth* and *form*. Music, then, can be seen as an organic process with a temporal signature that can be translated rather easily in terms of *movement*—both manifest and virtual—as advocated already in the seminal contributions by Truslit (see below). In the same vein, it is possible to externalize a felt experience by moving along with the music or simulate it merely in the imagination. Such “ideomotor” simulation (see below for the term) recalls the older “motor theory” of speech perception (Berthoz, 1997; Jeannerod, 1994, 1995; Scheerer, 1984; Viviani & Stucchi, 1992; Galantucci et al., 2006; and Reybrouck, 2001 for a musical analogy), which originally stated that the ability to simulate in imagery the movements needed for the production of sound while merely listening to speech makes it possible to conceive of it in motor terms without actually executing the sound-producing movements themselves. The theory, which originally focused on speech perception (Liberman et al., 1967; Liberman & Mattingly, 1985), was received rather critically at first (see Sussman, 1989). Later, however, it was also applied in domains of increased generality and gained more positive recognition (Rizzolatti & Arbib, 1998; Williams & Nottebohm, 1985), particularly through the discovery of so-called *mirror neurons* and the finding that motor imagery is also involved in perception (Gallese & Goldman, 1998; Gallese et al., 2007; Molnar-Szakacs & Overy, 2006; Overy & Molnar-Szakacs, 2009; Rizzolatti & Craighero, 2004). Perception, in this view, has access to the motor system, with perception–motor links that are working both at the neural and behavioral level (Galantucci et al., 2006).

The findings on mirror neurons, though still controversial to some extent—especially with regard to the generalization of applicability from animal research to human beings—have flourished in the past decade but are now witnessing some decline. Yet their propulsive drive is not extinguished but evolving (see Bonini et al., 2022 for a state of the art). What we want to claim in this paper, however, goes beyond the mere simulation or re-enacting of sound-producing gestures by applying the motor imagination not merely to those gestures that produce the sounds but also to the internally felt motor experience of the body as a whole. It is thus perfectly possible to listen to music without any visible trace of movement while at the same time having the feeling of being moved. Listening, then, can be considered in behavioral terms as holding a dynamic tension between outer (manifest) and inner (imagined or proprioceptively felt) movement, between moving and being moved, echoing somewhat the earlier claims by Alexander Truslit in his seminal book *Gestaltung und Bewegung in der Musik* (Truslit, 1938). The basic ingredient of music, in his view, is not so much sound as movement, or, as Sessions puts it: “[m]usic is significant for us as human beings principally because it embodies movement of a specifically human type that goes to the roots of our being and takes shape in the inner gestures which embody our deepest and most intimate responses” (Sessions, 1950/1971, p. 19)

### 1.2. Music as Movement Through Time

**Figure 1:**
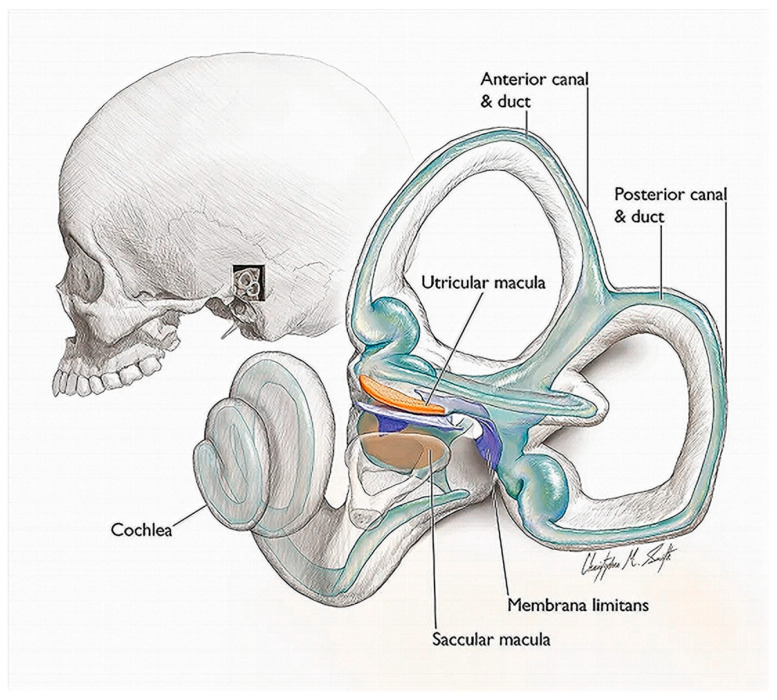
Schematic depiction of the anatomy of the hearing system with a left lateral view of the inner ear within the human cranium and an enlarged picture of the bony labyrinth with the membranous labyrinth inside. (Adapted from C. Smith et al., 2022, Copyright © 2021, American Association for Anatomy, Creative Commons CC BY).

Truslit’s ideas are highly relevant to contemporary attempts to understand the nature of musical motion and its communication through performance. They have a strong biological character and anticipate by several decades modern developments in psychology and psychomusicology (Repp, 1992b, p. 277) by stating that the “dynamics” and “agogics” of music (see below for the terms) provide the auditory information for motion perception. One of his major claims is that the *vestibular system*—the central part of the labyrinth in the inner ear, involved in balance of the body—is the organ of musical motion perception (see Figure 1). The idea is surprisingly modern and has even been taken up and restated by contemporary scholars (Todd, 1992, 1993, 1999, 2001; Todd & Cody, 2000; Todd et al., 2000; Todd & Lee, 2015; Todd et al., 1999) who also pursue the question of how to characterize natural motion in musical performance (Feldman et al., 1992; Repp, 1992a). There are some resemblances with the claims by Clynes and Gabrielsson, though the latter focus more narrowly on the rhythmic level of moving along with the music, while Truslit focuses more specifically on the movements of the limbs (Clynes, 1977, 1983, 1986; Clynes & Nettheim, 1982; Gabrielsson, 1986, 1988). Enticing as this theory may be, however, it has been the subject of considerable controversy (Repp, 1992b), as it was claimed initially that head movements and active stimulation of the vestibular apparatus were necessary to observe the expected effect, suggesting that the vestibular influence is not a direct one (Phillips-Silver & Trainor, 2007; Trainor, 2007; Riggle, 2009; Trainor & Unrau, 2009).

A lot has happened since Truslit’s early claims. There has even been a real revolution in vestibular research, with some kind of revival of his vestibular hypothesis in the early 1990s (see Todd & Lee, 2015). Several key developments have been influential in this regard: (i) the insight that the vestibular system is of primary importance to the perception of rhythm and that it is also dependent on the body, (ii) independent advances in imaging techniques that have identified the brain areas that are associated with both vestibular processing and rhythm perception, and (iii) vestibular receptors that have been found to contribute to auditory evoked potentials.

Starting from the findings of Tullio and Tait that the vestibular apparatus could respond to sound and that not all hearing is cochlear (Tullio, 1929; Tait, 1932), new investigation into vestibular sensitivity has been performed in auditory research with the discovery of myogenic—originating in muscle tissue—and vestibular-evoked responses that were referred to as the *vestibular evoked myogenic potential* (VEMP). This should be seen as a manifestation of a vestibular reflex mediated by acoustic sensitivity of the otolith organs—the otoliths are very small crystalline pieces of bone-like material found in the inner ear and the vestibular apparatus—and the spinal tract (Bickford et al., 1964; Townsend & Cody, 1971; Colebatch et al., 1994). The VEMP has since been used both as a non-invasive clinical tool for the diagnosis of vestibular function and as a clinical tool that could also be used as a scientific tool to investigate the acoustic sensitivity of the otolith organs to both air (AC) and bone-conducted (BC) sound. A major breakthrough was the finding that VEMPs could be activated by “sounds” from the environment, which clearly showed that the auditory and vestibular pathways are much more intertwined than hitherto suspected. It led scholars to study the vestibular influences on auditory rhythm perception (Trainor et al., 2009) and to formulate the *sensory–motor theory* of rhythm and beat induction, which states that rhythm perception is mediated by the conjunction of auditory input and motor representation of the body and that a sense of motion is mediated by the vestibular system (Todd, 1992, 1993, 1994, 1995). This basically means that rhythm perception is vestibular in the sense that brain areas associated with rhythm perception are closely correlated with the vestibular–motor network, or, stated differently, vestibular inputs, either from acoustic, gravitational, or inertial stimuli, are strong facilitating factors in the interpretation of auditory input. It is an approach that argues against more general and cognition-based approaches that are basically “disembodied” in favor of an “embodied” approach that restores to some extent the concept of self-motion as advocated already by Truslit (see below). The phenomenon of beat induction—the cognitive skill that allows listeners to hear a regular pulse in music to which we then can synchronize—can then be seen as a form of externally and internally guided action in sensory–motor circuits, with the vestibular inputs figuring in a privileged manner. Not all music is reducible to metrical rhythms, however. This is the case when the music does not have a beat—as in music that “flows” in a more organic way—but, even in that case, it is possible to sway the whole body along with the larger motions that are related to the phrasing of the music, and that can be considered a form of vestibular self-stimulation that may be created either through indirect associative links of sound shapes or direct vestibular activation (Todd & Lee, 2015).

The findings echo somewhat the early claims of the *motor theory of rhythm* (Stetson, 1905), which drew a link between gesture in movement and phrasing in music, but it was above all Truslit who gave the link between music and motion a fully articulated theory (Truslit, 1938). Central in his approach is the recognition of an intimate experience of music, which involves the sense organs for the perception of movement, the sound signal itself, the muscular sensations that are evoked, and the shaping of movement. This means that we perceive music not only with the ears but that our whole body and our whole being are absorbed by the music and by what he called the “experience of moving life.” He thus broadened the concept of movement not only to our own body but also to the broader conception of our body as part of life, which is characterized by its dynamic signature of being in continuous movement. All of this happens externally as well as internally, and even if this is barely noticeable, it may grasp us as a whole and lead to a lively musical experience (Truslit, 1938, p. 58).

Truslit’s recommendations, furthermore, concern those who “make” the music sound as well as those who “receive” it. They reflect the results of uncovering and utilizing the biologically determined sources and foundations of music, not as a collection of performance rules but as a profound reflection on education and the abilities of the senses to create and experience music from within. Listening, then, is a kind of inner sense of shaping (*ein inneres Mitgestalten*) freed of the technical problems of performing but implying an active way of listening. Its major effects come from the overall appearance of the tones—pitch, timbre, loudness, and duration—but most of all from its movement that takes on a particular form of progression. There is, as such, an inner connection between music, movement, and experience, which can be summarized in four major points:

- The expression of the musical experience is not limited to the experience of pure movement; there are even more differentiated expressions that can be expressed by a characteristic movement;
- This differentiated experience is also experienced through the mediation of the acoustic form of this movement;
- The special form of movement forms the bridge from the performer to the listener;
- Just as movement is not only a means of expression but also an essential part of emotional movement, movement in music is not just a means of creation but above all its most fundamental component (Truslit, 1938, p. 51).

### 1.3. More on the Vestibular–Motor Coupling and Acoustic Stimulation

Truslit’s claims are still thought-provoking. They could even function as research questions for current research (see below) in the sense that they open up perspectives to connect the experience of music with motion and affect, not merely in a metaphorical sense but also in terms of biological grounding. An issue that continues to raise questions, however, is whether *vestibular–motor coupling* works only in the case of effective movement while listening to music or whether mere acoustic stimulation is also a sufficient cause for its elicitation. We are inclined to answer in a positive sense, as it has been shown that rhythm perception evokes the same areas in the brain as those involved in motor timing, even when there is no explicit movement (Schubotz, 2007; Schubotz et al., 2000). The idea has remained controversial, however, as most of the empirical evidence was found in the case of actively moving along with the music (Phillips-Silver & Trainor, 2005, 2007; Trainor et al., 2009). Yet, the potential functional significance of the “efferent” vestibular system—efferent neurons carry impulses outwards to the effector organs; afferent neurons carry them towards the center of a body part—is still somewhat unclear, with several overlapping hypotheses that are based on the vestibular function of modulating “afferent” sensitivity. The overall picture is quite complex, with the vestibular nuclei receiving input from an array of cortical, cerebellar, and brainstem structures and vestibular involvement found in the vestibulo-sympathetic pathway, which has a role in altering the sympathetic efferent discharge of the autonomous nervous system (Metts et al., 2006; Holstein et al., 2014, 2016; and Mathews et al., 2015 for a broader overview).

There is no space to elaborate in depth on the specifics of the vestibular function (see the relevant literature). If we were to summarize a little, it can be stated that the *vestibular system* is an evolutionary old system that provides the sensory end-organs—the peripheral vestibular system (PVS)—for capturing the gravitational frame of reference for many biological processes. It is fundamental for how we perceive ourselves in three-dimensional space by relying on the fluid-filled membranous structure (the membranous labyrinth) that is housed within a bony shell (the bony labyrinth) that is located in the temporal bone of the skull (see Figure 1). It includes five paired organs, namely, three *semicircular canals* with fluid-filled ducts and two *otolith organs*, the *utricle* and the *saccule*. The semicircular canals detect angular acceleration of the head, while the saccule and the utricle detect changes in horizontal and vertical linear acceleration. They sense vibration and head tilt due to the deformation of the hair cells that are weighted with small stone-like crystals or “ear-stones” (from the Ancient Greek: οὖς (oûs) = ear + λίθος (líthos) = stone) (C. Smith et al., 2022, 2023).

The vibrational aspect in particular requires additional research, which is not obvious given the inaccessibility of the vestibular system that is embedded in the hardest bone (the petrous bone) of the temporal bone. Quite promising is the finding that there is a link through the ductus reuniens between the peripheral hearing system and balance mechanisms and the major role therein of the otolith system. What is needed, in this regard, is an in-depth study of the electrophysiology of the vestibular system to analyze the relationship between hearing and movement. Studies on dancers have already been revealing to some extent, showing that stimulation of the auditory system also stimulates the vestibular system, thus pointing in the direction of thorough sensory–motor integration (de Oliveira et al., 2022).

In what follows, we go into this more deeply by elaborating on the delicate tension between inner and outer movement, which could be described also in terms of a “centripetal” and “centrifugal” approach to music listening. There is, in fact, a distinction to be made between the sensory information that is picked up by the senses, with a possible resonance within the body (centripetal), and the externalization of our responses to these stimuli that find their way back from the inside to the outside (centrifugal).

## 2. Music as Inner and Outer Motion: Moving and Being Moved

“In the beginning was rhythm” (*Im Anfang war der Rhythmus*) is a much-quoted phrase by Hans von Bülow. According to Truslit, we should even add movement to the term, as the original meaning of its Greek origin—ῥυθμός—means to flow, uniform movement (Truslit, 1938), or lively flowing (Becking, 1928). Over time, however, this broader meaning has shifted from a particular kind of flowing to a rather inorganic accentuation that does not do justice to its grounding in movement. Natural movements are continuous and not discrete. They entail an element of fluency and goal-directedness that may give them some telic potential. As such, there is a distinction between mere “flux” and “continuity” in the sense that continuity also involves forces and structures that endure through change (Dewey, 1934/1958).

This is even more the case with music that seems to follow a *trajectory in time*. The analogy with the *river metaphor*, therefore, is obvious, as a river moves through time and space; it is forever changing and moving; it flows sometimes slowly, sometimes very fast; it flees freely, and, even if it meets varying degrees of hindrance in its course, the movement of the mass of water flows from source to mouth. As Dewey puts it: “A river, as distinct from a pond, flows. But its flow gives a definiteness and interest to its successive portions greater than exist in the homogeneous portions of a pond. In an experience, flow is from something to something. As one part leads into another and as one part carries on what went before, each gains distinctness in itself. The enduring whole is diversified by successive phases that are emphases of its varied colors” (Dewey, 1934/1958, p. 36).

**Figure 2:**
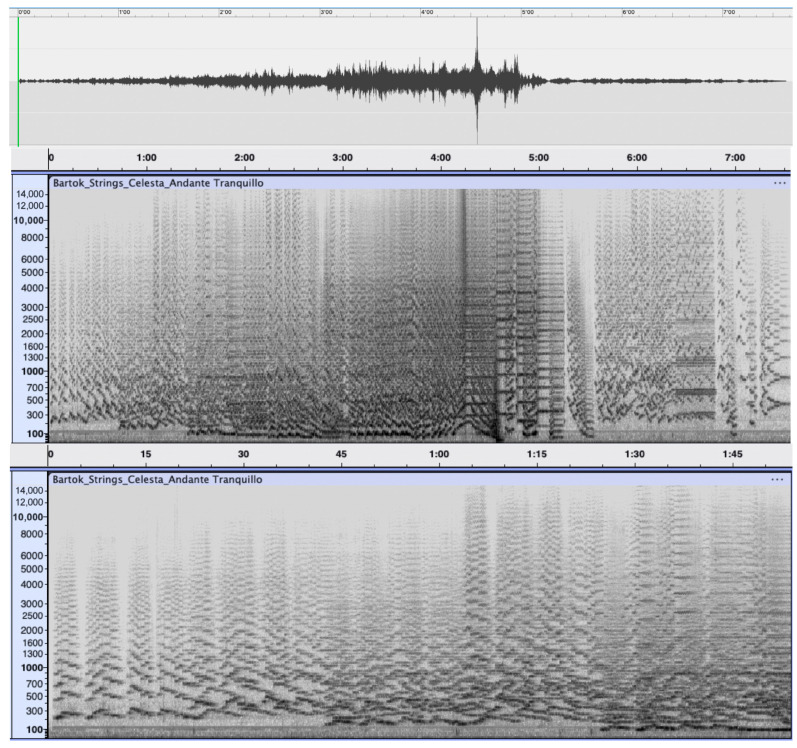
Visual representation of the first movement of Béla Bartók’s *Music for Strings*, *Percussion and Celesta*. The upper pane shows the waveform representation, the middle pane shows the spectrogram, and the lower pane is a zoomed-in version of the first 1.45 min (Copyright © 2025 by the author).

It is challenging to translate this to the realm of music, which, as a moving sonorous mass, also describes a trajectory through time and imaginary space. The translation, however, is not unequivocal, as it is not obvious to describe the flowing of such an immaterial thing as music. A possible answer lies in the definition of music as a *fluidum,* with some initial inspiration to be found in the branch of physics that deals with the deformation and flow of materials and that is commonly known as *rheology*. Before delving into the technicalities of the field, we first present a musical example in Figure 2 to indicate in which direction music listening could be understood as a kind of “applied rheology.” The figure shows the first movement of Béla Bartók’s *Music for Strings*, *Percussion and Celesta*. The fragment has a duration of 7.38 min and is depicted in a highly synoptic and compressed way as one single image, both as a waveform (upper pane) and a spectrogram (middle and lower pane), which represent, respectively, the time domain (waveform) and the frequency domain (spectrogram) representation of the musical signal. The waveform (upper pane) has a high temporal resolution and clearly shows the dynamic development of the sonorous volume of the music, starting from a low level of sound energy to a high around 4.30 min, to decrease again to the end. It clearly illustrates the teleological dimension from start to end, with something happening along the way to the final decay. It is a representation that can be seen with the naked eye and that provides a kind of *macroscopic* view of the musical progression. The spectrogram (middle pane) has a lower temporal resolution, but, by showing the individual frequencies of the vibrations, it provides a more *microscopic* view of the spectral content of the music, allowing us to follow the temporal courses of pitches of the music rather than its sounding energy. Zooming in (lower pane) makes their individual trajectories over time even more visible. It shows the additional value of using visualization tools, which make it possible to conceive of music not only in *rheological* but also in *microrheological* terms. As will be shown further, it can be argued that skilled listening aims to emulate this kind of high-resolution processing of the sounds (see below for an explication of the terms).

Care should be taken, however, not to confuse the visual representations of the waveform and the spectrogram, which are static images, with the music that evolves over time. There are, however, visualization tools that make it possible to look at the visualization with the help of a moving cursor that keeps step with the sonorous unfolding. This can give a first glimpse of the experience of so-called rheological listening, and it can be assumed that some training with these moving visualization tools can lead to the mastering of the skill of having this rheological experience even without the help of these visualizing tools.

### 2.1. Music as Fluidum: Rheology and the Study of Matter That Flows

Describing music as matter or substance that flows makes it possible to conceive of it in “rheological” terms and to extend the analogy between the way music unfolds in time and how a fluid flows in a duct. It is a challenging approach that aligns the study of music with the study of fluids, with the aim of capturing the elusive nature of a dynamic equilibrium between states at certain moments in time (Guerra-Valiente, 2024).

Rheological concepts have been studied throughout the ages when dealing with flowing materials, but the establishment of rheology as an independent branch of natural sciences was formally introduced by Bingham in 1929 at a Plasticity Symposium on the study of viscosity. This was the start of the development of its disciplinary history, with a name that was chosen to refer to Heraclitus’ quote that “everything flows” (τὰ πάντα ῥεῖ) (Wilson, 2018). Its main focus was inspired by observations of strange and abnormal behaviors of known materials, such as paints, clay, yogurt, concrete mix, polymers, pharmaceutical pastes, sealants, and others, that exhibit a mixture of liquid-like and solid-like properties. Paints, for instance, can be poured into containers, but they stay on vertical surfaces without sagging down; clay looks solid but can be molded into shape; yogurt does not flow out of a container when held upside down but demonstrates reduced viscosity after mixing. Other substances, such lubricants, pastes, sealants, gels, and even body liquids (saliva, mucus, sperm), behave as liquids but become solids that remain on the surface to which they are applied. These examples are enlightening, but they clearly illustrate the insufficiency of dichotomous categorizations in terms of solids vs. liquids (Malkin & Isayev, 2005), hence the need to expand the descriptive and explanatory vocabulary for the study of the field.

Rheology, which, in the broadest sense, is the science of the description of the flow behavior and deformation of all types of matter (Bingham, 1944; Walters, 2010, p. 92; Doraiswamy, 2002), envisioned originally the theory that underlies this deformation and the practice of its measurement (Wilson, 2018). This broad definition, however, does not emphasize sufficiently some of its major features; rheology is not only about deformation and flow but also about properties of matter that define its behavior as a reaction to that deformation and flow. It studies properties of materials that are described by any relationship between force and deformation, with an emphasis on non-linear dependencies; the deformation of the studied materials results in the superposition of viscous and elastic effects, and the structure of the materials changes under the influence of the forces applied to them (Malkin & Isayev, 2005).

As such, there have been fruitful attempts to provide operational definitions to describe the rheological behavior of real or virtual continuous media. Most fruitful is the concept of *fluidum*, which can be defined as a substance (liquid or gas) whose particles can move about freely. Three major points might be considered here: (i) the use of different time scales while observing flowing behavior, (ii) the description of forces that act on materials, and (iii) the actual behavior of distinct real continuous media.

(i) The particular time scale of description, first, is important in the sense that the results of observations of liquid- and solid-like features reflect changes in material structure that take place during the period of observation (Malkin & Isayev, 2005). It is an important measure of the ratio of the time rate of inherent processes in an observed material to the time of the experiment or observation. It has a quantitative expression in the *Deborah number*, which is a non-dimensional number that finds its origin in an Old Testament scripture from the Book of Judges: “The mountains flow before the Lord” (Chapter 5, verse 5). It means that everything flows, if we just wait long enough. Deborah thus knew two things: the mountains flow, but a human in their short lifetime cannot see them flowing, whereas the time of observation of God is infinite. The Deborah number (D), then, is the ratio of a characteristic relaxation time of a material to a characteristic time of observation of the relevant deformation process (Reiner, 1964). The idea is fruitful in its simplicity, but it has a lot of operational power. It simply means that the magnitude of D defines the difference between *solids* and *fluids*. When the time of relaxation of the material under observation is short, the material is perceived as flowing; it the relaxation time is larger than the observation time, the material appears as a solid. The Deborah number, therefore, is a fundamental number in rheology, as it subsumes solids and fluids under one common concept, with a greater D referring to solids and a smaller one to fluids (Walters, 2010).

In a first attempt to translate Deborah’s number to the realm of music, we refer again to Figure 1, where the middle pane—the spectrogram of the whole movement—provides a synoptic view of the whole movement, with the listener acting as a still spectator who oversees the whole picture at a glance, while the zoomed-in version of the lower pane limits the temporal window through which the listener has access to the selected zoomed-in portion of that window. The distinction actually only defines the *level of resolution*, with the lower resolution of the synoptic view referring to a rather static representation of music, while the higher resolution provides a more dynamic and fine-grained mode of tracking the sound.

**Figure 3:**
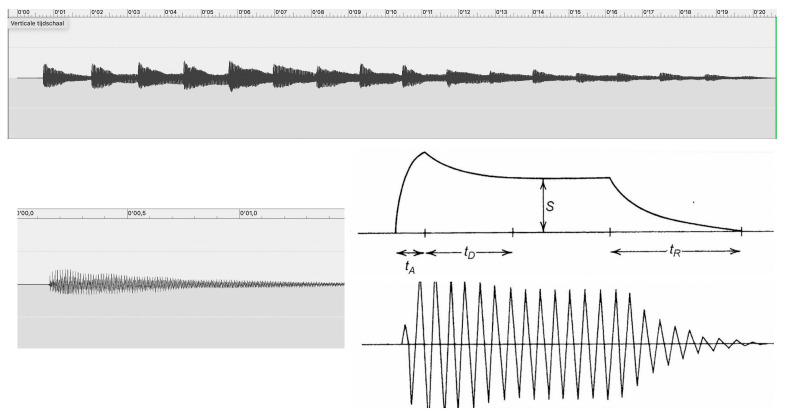
Waveform depiction of the first 16 notes of *La grande prière* by Georges Gurdjieff (upper pane) and a zoomed-in version of the first sound (lower pane, left) and its comparison with an ideal ADSR envelope of an acoustic sound (lower pane, right). (Copyright © 2025 by the author).

The reference to the Deborah number may seem a bit naïve at first sight, but its intuitive appeal makes a lot of sense. Let us take the example of piano sounds, which are, in fact, percussive sounds. Pressing a key causes a felt-covered hammer to hit the tuned strings that are located in the soundboard of the piano. The use of felt makes the sound a little less percussive, though it remains, ultimately, a percussive sound. Being an acoustic sound, however, it is also characterized by an ADSR envelope, which stands for four stages of modulation of its intensity: *Attack*, *Decay*, *Sustain*, and *Release*. It is the most natural course of the generation of a sound that takes the shape of a waveform with four distinct stages: a rising during the attack stage, a slight decline during the decay stage, some plateauing during the sustain stage, and returning to the original stage during the release stage. Figure 3 provides an example. It depicts the first bars from *La grande Prière* by Georges Gurdjieff, showing a succession of single piano sounds, which can be labeled as discrete pitches (corresponding to F4). The upper pane shows 16 separate ADSR envelopes with decreasing intensity. Zooming in on the figure clearly shows that each separate ADSR curve can be considered as a discrete event but also as a dynamic trajectory through time, which is continuous. The lower pane shows the zoomed-in version of the first sound (left), which aligns pretty well with the standard ADSR curve (right). It is a beautiful example of the transition from something discrete—the separate sounds—to something that flows. The analogy with the Deborah number, moreover, is also clear. The broader view (upper pane) presents the individual sounds as relatively stable and unchanging (the relaxation time of the music is much longer than the momentary synoptic view of observation), while the more limited temporal window of the sounds (lower pane) shows the discrete event in its temporal unfolding, thus raising the time of observation vs. the time of sounding, giving it some rheological signature that makes the music more fluent. One may even wonder whether the distinction between a solid and a fluid is looming here, in the sense that the lower resolution materializes the sound as a solid (upper pane), while the higher resolution evokes the image of a fluid (lower pane). It can be argued, in this regard, that equating the time of relaxation and the time of observation provides an operational definition of *sound tracking*, in which case the listener follows perceptually the rate of the temporal unfolding, thus experiencing the flowing character of the music as a fluidum. This should mean that we give the Deborah number the value of 1.

(ii) The description of forces, second, is related to the mechanical properties of the flowing materials. These can differ greatly from each other with, among others, rigid solids for which only the mass is important, as they do not undergo deformation; elastic solids, with stress being proportional to strain (see below for the terms); inviscid fluids, which exhibit no resistance to flow and where viscosity effects are mainly absent; and Newtonian fluids, where viscosity is the resistance that arises from the lack of slipperiness originating in a fluid (Doraiswamy, 2002). All of these flowing materials can be described quantitatively in mathematical models that represent the rheological properties of all types of matter in terms of a relationship between forces acting on materials and changes in their shape (Malkin & Isayev, 2005). It is then possible to describe their rheological properties in terms of stress, strain, and their mutual ratios and quantify them by using constitutive equations between the stress history and strain history of these materials (Doraiswamy, 2002). *Stress* refers to the amount of force that acts on materials, whose responses are quantified in terms of the amount or rate of deformation. This is the *strain*, which appears when layers of material slide over each other (shear strain) or when a change is created by extension —elongation or shortening—in one direction of the material (normal strain). Their mutual ratios define the elastic modulus for a solid (stress to strain ratio) and the viscosity or flow rate for a liquid (stress to rate of strain). Many materials, moreover, have liquid as well as solid features, which means that their elastic moduli and viscosities are not constant magnitudes but functions of time, force, and the direction of the forces applied to them (Janmey & Schliwa, 2008).

(iii) The actual behavior of continuous media, finally, concerns their flow behavior, which can be described as a relationship between external actions, such as forces applied to the flowing matter, and internal reactions of the matter, such as changes in its shape. The mathematical formulation of this relationship between forces and deformation is subject to some constraints: there is a need for a point of reference and some arbitrary points in the matter by measuring, e.g., the changes in distance or angles between two points; it is assumed that there is no discontinuity in the transition from one point to another; the mathematical analysis of infinitesimal quantities can be used; and the changes occur gradually without discontinuities (Malkin & Isayev, 2005).

It can be asked, finally, how to translate this to the realm of music. There are indeed many metaphors that refer to the flowing character of music as a temporal and sounding art, but there is still a need for operational descriptions of music as a fluidum and a characterization of possible rheological features of the sonorous articulation over time. A valuable attempt in this regard has been made by Guerra-Valiente, who tried to merge music, fluid mechanics, and drawing by fitting it into a rheological model that was inspired by Deleuze and Guattari, who distinguish smooth and striated space (Guerra-Valiente, 2024; Deleuze & Guattari, 2004). This would have the inertial and cohesive forces as fundamental properties of a musical composition considered as matter that flows. It was primarily related to the notion of continuity and discontinuity and the many ways musical space and time can be divided (see also Campbell, 2013) and has been translated to the domain of music by Pierre Boulez (Boulez et al., 2019, p. 18).

### 2.2. From Flux to Continuity: Music as an Organism

The rheological description of music as a fluidum is an interesting starting point. It can be reductionistic, however, in conceiving of music as an inanimate flowing object. One may ask, therefore, what is the distinction between the water of a mountain river that seeks its way by meandering through the landscape and the musical flow as an articulation over time. The flow of the water seems to depend mainly on external circumstances, such as the hardness of the soil or the obstacles it encounters in its course, which means that its trajectory is externally driven and not driven by some inner force. Taking a broader perspective, however, one may add a telic dimension in the sense that there is a flux from a starting point to a further point, which might be perceived as an element of growth and form. This is the transition from *flux* to *continuity*, which, according to Dewey, is the hallmark of nature and life. Continuity involves forces and structure that endure through change, and they do so more slowly than surface accidents. As such, they are relatively constant. The changes, on the other hand, are not all gradual but may culminate in sudden mutations and transformations that, taken from a later perspective, may have their place in a logical development (Dewey, 1934/1958, p. 323).

The concept of continuity challenges the inanimate character of a flowing object. It appeals to the distinction between living and non-living things, which starts early in life. According to Pinker, “[i]t initially takes the form of a difference between animate objects that move around according to the laws of billiard-ball physics and objects like people and animals that are self-propelled” (Pinker, 1994, p. 423). The major question, then, is whether music should be considered an animate or inanimate object. For Coker, music has the gestural force of resembling objects, animate or not, for which we can adopt the root metaphor of the sonorous object as a sound-producing “organism” (Coker, 1972, p. 3). The metaphor received some attention in earlier writing (Reti, 1961; de Sélincourt, 1920), with attempts to “hypostasize” the music as a living organism, though this has been criticized as being too metaphorical and not sufficiently operationally testable. The idea, however, has been taken up again in recent empirical studies on musical agency (Schiavio et al., 2023) and interactive dynamics as applied to music (Reybrouck, 2023).

The metaphor of *music as an organism* is useful to the extent that it places music and life under a common heading. Both are experienced as dynamic processes of growth and decay, activity and rest, and tension and release, with the processes being differentiated not only by their course and shape but also by the quality of the motions that are involved in them (Meyer, 1956/1961, p. 261). A similar approach can be found in Hartmann’s description of the world of sound, which he describes in rather romantic terms: everything is movement, excitement and calming, surge and swelling, subsiding and fading, quiet murmuring and whispering or dark rumbling, wild roaring, storming, escaping and chasing, as well as taming of unleashed forces in the music’s form. All of these processes must be absorbed in the temporal flow and movement and should be able to trace the dynamics of our mental processes (Hartmann, 1953, p. 204).

The comparisons are appealing, even if they lack the rigor of scientific explanation. They emphasize the dynamic aspect of development over time, which can be described in terms of growth and form, which make up the title of d’Arcy Thompson’s seminal book—*On Growth and Form*—and must be understood in terms of their relationship to the study of organisms. Growth, in D’Arcy Thompson’s view, must be studied in relation to form, either as a simple increase in size without obvious alteration of form or as a gradual change of form and the slow development of a resultant structure. Both can be explained by physical considerations or described in mathematical formulations (D’Arcy Thompson, 1917/1942, p. 15). The concept of form then takes on a dynamic signature, and such an “organic” form—or morphology, as Goethe coined it—is only a portion of the wider science of form, which deals with all possible forms that can be taken by matter under all aspects and conditions.

**Figure 4:**
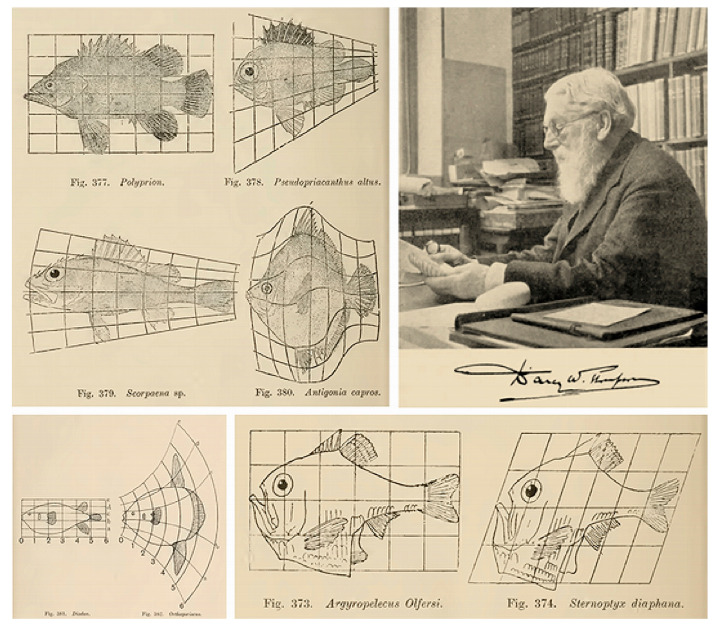
Examples of some famous transformations of fish shapes in D’Arcy Thompson’s seminal book by using his method of coordinates, with resulting transformations. (Reproduced from Wolfram, 2018, Copyright © 2018, Springer Science Business Media, LLC, part of Springer Nature, License Number 6087170899089).

Goethe’s approach is fascinating, especially in an age of static classifications. It has been influential in the context of evolutionary developmental biology, but it can also be somewhat confusing, as he defined the concept of morphology both in a process-oriented and dynamic way of thinking, combining both form as Formation and form as Transformation (see Toni, 2018 for an in-depth discussion). Morphology, in that broader view, is not merely the study of the forms of material things, as it also entails a dynamical aspect that goes beyond a description in static terms in favor of an understanding of the forces that give rise to it. These forces can be considered forces in equilibrium or forces whose magnitude and direction convert one form into another (D’Arcy Thompson, 1917/1942, p. 1027). Their description may be produced in terms of common language, but it is also possible to use the more precise language of mathematics by using the *Method of Coordinates* on which the *Theory of Transformations* is based. It is a method that makes it possible to compare or recognize one form as a permutation or deformation of another, as in the mathematical Theory of Group, with a distinction between substitution groups and transformation groups. The former are discontinuous—one form is substituting another—and the latter are continuous, with only infinitely small differences from each other (D’Arcy Thompson, 1917/1942, p. 1032). Figure 4 provides an example of some of the most famous transformations, as illustrated in his seminal book.

The figure clearly illustrates how deformation can work by first drawing a form over a rectangular grid and then performing a single transformation of the grid structure by bending the lines in a systematic way. It was used to study the coordinate transformations of the forms of similar animals, but the method has multiple applications through its broader mathematical visualizations in 2D or 3D, and it is even challenging to conceive of a possible application in the visualization of music as something that develops over time.

**Figure 5:**
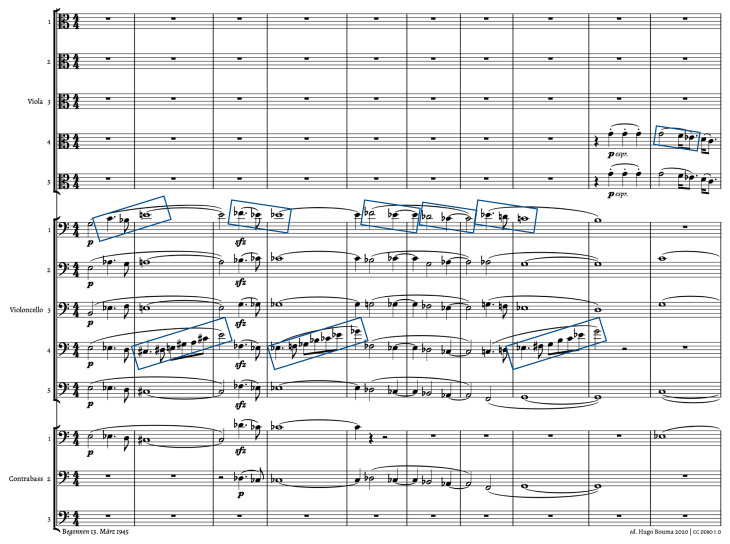
Score notation of the first measures of Richard Strauss’ *Metamorphosen for 23 strings*. Some of the motifs are repeated under continuous modification (highlighted), giving an impression of topological transformation (Copyright © 2025, IMSLP-Pubic Domain).

It can be questioned, however, whether the translation from visual to musical transformations makes sense, as visual and auditory processing rely on different areas and functions in the brain. We are inclined to answer positively if we are ready to rely on visualization tools to make the sounding music visible. Waveforms and spectrograms are obvious examples that provide a rather continuous representation of the music, but even a symbolic representation, such as a traditional musical score, can be helpful here. Figure 5 provides a first example. It depicts the first bars of Richard Strauss’ *Metamorphosen for 23 strings*, which is an iconic example of flowing energy, with small patterns that are repeated while undergoing continuous transformations. The ascending melodic motif in the fourth cello is repeated three times, but always in an ever-changing form that retains the general melodic and rhythmic shape but with different pitches. The other celli also show short melodic and/or rhythmic patterns that are repeated under transformation. The overall picture is one of an interwoven musical texture with short pieces of musical material that behave as transformations of each other and thus generating a kind of continuous flux, with the ending of one coinciding with the beginning of another. One could imagine this flux as a kind of sonorous mass that undergoes topological transformations (either in 2D or 3D or even in more dimensions), with inspiration from the domain of *topology*, which is also known as the “rubber sheet geometry” as the branch of mathematics that studies the properties of shapes that remain unchanged under continuous deformations. This means that shapes can be stretched, twisted, bent, or shrunk but without being torn, glued, or having holes that are created or removed, hence the comparison with a sheet of rubber that can be manipulated without breaking it or creating new edges.

### 2.3. The Concept of Organic Form and the Notion of Process

The dynamic approach to music can find a lot of inspiration in the concept of *organic form*. It was introduced by Coleridge, who contrasted organic with mechanic: “The form is mechanic when on any given material we impress a pre-determined form, not necessarily arising out of the properties of the material—as when to a mass of wet clay we give whatever shape we wish it to retain when hardened. The organic form on the other hand is innate, it shapes as it develops itself from within, and the fullness of its development is one and the same with the perfection of its outward Form” (Coleridge, 1914, pp. 46–47).

The concept has been very influential, particularly in the domain of biology, but also in the mathematical domain of topology and even in the domain of music (Schmidt, 1990). As such, it aligns with the idea of *morphogenesis* rather than *morphology*. The latter, as a general term, describes an order of reality that is characterized by terms like position, form, change, discontinuity, differentiation, stability, regulation, organization, etc. (Petitot-Cocorda, 1985, p. 73). Morphogenesis, on the contrary, designates any creative or destructive process of form without worrying about either the nature of the substrate of the forms or the forces that cause these changes (Thom, 1972, 1980). Being a conception with a biological and vitalist signature, the term morphogenesis found its origins in natural philosophy and the dynamicism of German speculative idealism. It thus influenced the history of biology to arrive through Driesch’s entelechies—a term borrowed from Aristotle’s conception of final cause to describe an organism’s capacity to regulate organic development—at Waddington’s concepts of “morphogenetic fields” and “chreodes”—a term used to visualize the ways in which evolution progresses through canalized paths of development (Waddington, 1942, 1962, and Petitot-Cocorda, 1985, p. 27 for an in-depth description of the terms). What matters in this “telic” approach is a strong departure from the tacit adherence of Western philosophy to static spatiotemporal and physical forms of order in favor of the notion of *process* (Whitehead, 1929/1957).

This notion of process has also been adopted in much theorizing about music, mostly in connection with the above-mentioned concept of *organic form*. Musical form, in this approach, should not be reduced to some static description as a formal moment but rather conceived as the awakening and entertainment of the listener’s feelings, to use Michaelis’ original conception (Michaelis, 1795, 1801). It is an approach that opposes the prevailing “tectonic” and “geometric” conceptions of form by valuing a “kinetic” over a “static “approach, typically exemplified in the writings of Kurth and Assafjew (Kurth, 1925, 1931; Assafjew, 1976). Both scholars were contemporaries, with Assafjew being influenced quite strongly in his early career by Kurth, though he became more critical as he grew older. Their points of agreement revolved primarily around the basic concept of *energetics* and *dynamics*, but the way they dealt with these terms became gradually more divergent. For Kurth, energy was not in the first place a force present in matter but a tension of an inner, spiritual nature, with its emanation being regarded as sounding, acoustically perceptible music. For Assafjew, on the contrary, energy was not primarily a psychological or metaphysical category but a category grounded in the process of “intonation” (see below), with significantly more emphasis on the musical material itself (see Danuser, 1986–1987 for an in-depth discussion). Both approaches, however, are closely related to the concept of organic form.

Kurth, first, has delved deeply into the notion of *dynamic form*. Focusing mainly on the *large-scale structures* in the Germanic symphonic repertoire of the latter half of the nineteenth century, he has argued for a description of form as continuous processes of transformation of an initial motive throughout a work rather than aligning with the long-standing conception of form as architecture, as a kind of standardized set of prescriptions for sectional outlines of a major work. It is a way of conceiving of form as “forming” or “becoming” as something that is organically evolving, thus adopting a dynamic approach to the concept of musical structure. This concept of the musical work as a dynamic process—what he termed *formal dynamics*—was not original to Kurth, however. It echoes even earlier views by Herder and Rousseau and a lot of metaphorical descriptions that have unfortunately faced a lot of opposition in certain academic circles. Yet his much-used analytical terms (e.g., “intensification” and “wave”) have a lot operational power. They align well with conventional concepts, such as “development-motives” or “kernels,” which possess both some kinetic power and a strong developmental potential. The formal dynamic process, then, lies in the ordering and transformation of these kernels so as to realize the dynamic shape of a work as a whole. The concept of a wave, on the other hand, is a further extension and transformation of these kernels. It is characterized by an intensification to rise towards a peak over much longer spans—mostly as a melodic peak, but also reinforced by rhythmic, dynamic, and textural factors—to ebb or dissipate thereafter but often with other, new waves, starting again, resulting in a shaping play of waves. The main focus of Kurth’s critical attention, however, was on the moment to moment transformation of melodic motives, with some attention to rhythmic detail and orchestral texture as auxiliary aspects to be considered, as well (see Parkany, 1988 for a broader, in-depth discussion).

Kurth’s approach, while appealing, has been criticized, however, by contemporary readers who find his approach somewhat unsystematic and personal, relying on intuition rather than established methodology (Rothfarb, 1988). His abundant “metaphoric-figural language” can be questioned from a scientific point of view, but his “personal” approach still makes a lot of sense. Contrary to approaches that start from theoretical, style–historical, and other explanations, he takes as a starting point the psychic functions that primarily give rise to musical hearing. They make it possible to describe the music as experienced, not limited to the experience of individual moments in time but as the perpetual evolution of the listening experience that combines the perception of the moment to moment unfolding with impressions of past sounding material and prospection of what still has to come. Listening, then, is a complex process that allows for the immediate perception and remembrance of both time and space. This means that over time, the listener may absorb both the *succession* of concomitant impressions and their *unification* in a broader, overall picture. It allows for both the perception of motion and the formation of an image of it, which means that music listening starts with the embodied experience of a flowing force, but it is only over time that the listener develops a fuller sense of an image over the entire course of motion. Central in his approach are the intertwined, unconscious experiences of forward and backward hearing, which Kurth refers to as *multitrack listening*, implicating multiple strands of time. What is meant, then, is a psychological process that constitutes a double-track course of hearing with listening not only occurring in aural imagination but also as a sense of feeling energy. Or, stated differently, listening is not merely a reproductive process of hearing but a generative process of creative power, with perception being a productive act (*Erformung*) that underlines the participatory roles of both the listener and the performer (Kurth, 1931; see also Tan, 2017).

Assafjew, on the other hand, has argued for a conception of *musical form as processus* to describe the organization of musical movement. Music, in his view, is a system of organized movement rather than a loose and orderless succession of sounds. The system is described in his *theory of intonation*, which he defines as the organization of acoustical media by the human consciousness into meaningfully expressive sound correlations and that can be conceived as a constructive principle of any organic musical progression and operationalized in terms of *Initium*, *Motus*, and *Terminus* (Assafjew, 1976).

Every musical enunciation, in this view, can be considered a state of unstable equilibrium that is delimited by the impulse of the beginning and the closure of an ending point. It is summarized in the ternary formula (I:M:T), which embraces three major moments: Initium (I), which is the impulse or starting point; Movere (M), the movement that is in a state of unstable equilibrium through transmutations; and Terminus (T), which is the ending or termination as a return to equilibrium. There is, in sum, a dynamic tension between a motivated enumeration that represents the tendency towards an opening of the work and the resulting summation (see Stoianova, 1975, 1976 for an in-depth discussion). The I:M:T formulas, moreover, do not necessarily follow each other neatly, but they can be intertwined in complex ways, with the Terminus of one formula figuring as the Initium of another, thus resulting in a complex texture.

There is no space to delve into the music–analytical details here, as this is beyond the scope of this journal. A reference to contemporary theorizing about musical form is, however, in order (see Bergé, 2009). A representative scholar in this regard is Caplin, who states in general that most descriptions of form take as a starting point the segmenting of the music in distinct and contiguous time-spans at multiple levels in a structural hierarchy, relying on an analysis of the work’s grouping structure or chunks. The music is then divided into separate parts, mostly denoted by letters (ABA, ABACA, AAB, etc.) or other verbal labels. Such a “labeling” analysis, however, falls short in not representing in an explicit manner a fundamental aspect of form, namely, its intimate association with the temporal unfolding of the music. Put in simple terms, this means that the experience of time is characterized by perceiving something that has a beginning, a middle, and an ending. These temporal unfoldings can be conceived as general “temporal functions,” which can be extended with the additional framing functions of what occurs before the beginning and after the end. Musical form, then, directly engages our temporal experience (Caplin, 2009). It is an approach that reflects the unique temporal character of each time-span at the surface of the piece, which is also known as the “beginning-middle-end” paradigm of introversive semiosis (Kofi Agawu, 1991). The temporal functions, however, are more diverse than these simple labels might suggest. They have been termed *formal functions* by Caplin in an attempt to differentiate how such spans express their temporality. He espouses a “form-functional” analysis, with each formal function arising from criteria that involve multiple parameters (such as harmony, tonality, grouping, and cadence). A summary of these functions embraces the broad functions of the extended classical sonata form: before the beginning, slow introduction, exposition, initiation, development, recapitulation, coda, and after the end. Conceiving of these as “functions” has the advantage that they invite the listener to engage with musical time rather than merely applying labels—also called formal “types”—without temporal expression. Formal types are thus rather atemporal, where formal functions are associated with the experience of time (Caplin, 2009).

Music can thus be described in processual terms (Osborne, 1986). This then entails a dynamic signature of form that highlights the role of continuity, progress, and growth, as exemplified most typically in biological organisms. As such, it should be distinguished from a static conception of form or structure that neglects the genetic aspect of its coming into being. The concept of morphology, therefore, is not only the study of material things and their forms; it also entails an interpretation in dynamical terms of forces and energy that mold their final structure (D’Arcy Thompson, 1917/1942, p. 18). This can be considered at several levels of temporal scales, with the millisecond level at one extreme and the longer temporal spans at the other. The latter is typically exemplified in the full life cycle of an organism, which may comprise distinct phases—embryonic development, growth, aging, death, etc.—and may be subsumed under the broader term of *morphogenesis* (von Bertalanffy, 1968, p. 122). It is not difficult to apply this also to the life cycles of music—hence the metaphor of music as an organism—with a distinction between the short-lived instances of pulses and beats as used abundantly in popular music and the larger temporal spans that may last even for hours in some examples of the above-mentioned romantic classical music.

There is, finally, a distinction to be made between static and dynamic descriptions of form, which is somewhat related to the distinction between geometry and physics. There is no space to go into detail here, but the growing momentum of the emerging field of linear algebra as the branch of mathematics that studies vectors, vector spaces, and linear transformations, together with the field of topology that studies properties of spaces that are invariant under continuous deformation—the so-called “rubber-sheet geometry” (see above)—with objects that can be stretched and contracted, opens up a lot of perspectives for a more rigorous description of *morphodynamic transformations*. Much is to be expected here from the domain of *spectromorphology*, which studies the dynamic shapes, contours, and morphological characteristics of sounds, considered with respect to their spectral content. This reflects the temporal dynamics of sound that reveal its evolution and structure, shaped by articulation, internal coherence, and temporal changes (Smalley, 1997).

Unlike most visual forms that reveal their content at a glance, sound forms are inherently temporal. They manifest shapes that unfold dynamically and emerge from the relationships between their internal elements. Qualities like pitch, intensity, and spectral energy may contribute to the perception of form, but it is mainly the temporal dynamics of sound that reveal its evolution, offering insights into its organization, morphology, and unfolding nature.

The distinction between *simultaneity* of representation and *successivity* of the temporal unfolding is a critical distinction in this regard. It is a recurring theme in epistemology: paying attention to the structure of something is mostly at the expense of valorizing its genesis, its history, and its function. But structure and genesis are not necessarily opposed to each other. They are even intertwined in the sense that genesis is only the passage from one structure to another.

This genetic approach can be easily translated to the realm of music, which, according to Brelet, is created before our eyes: music is the temporal art par excellence that needs time to construct its form (Brelet, 1949, 1968). Unlike visual arts, which present their content in its simultaneity, music does this step by step. It grows by accretion in the sense that each new element is amended by the next into something that is close to the intended total meaning. There is, in other words, a build-up through stepwise change of the initial image (Arnheim, 1969, p. 250).

This tension between *simultaneity* and *successivity* has received a lot of attention in the epistemological and psychological literature. It has also played a major role in the writings of some early music psychologists. Kurth, as one of their principal exponents, has pointed out that memory-based imagination may generate the paradoxical appearance of a simultaneity staggered backwards in time. This refers to the psychological ability to hold together in an instant what unfolds in temporal succession and appears to be tainted with spatial impressions (Kurth, 1931, p. 96). A somewhat related idea has also been advocated by Meyer, who stated that a musical form achieves the double ends of *articulation* and *segmentation*, combined with *unification* and *forward-movingness,* by putting the various cues close to one another (Meyer, 1973). It is an approach that raises criticisms against those music theorists, such as Schenker, who tend to treat musical compositions as a thing rather than a process that gives rise to a dynamic experience (Meyer, 1956/1961, p. 54).

Kurth has developed these claims in depth in terms of *inner dynamics* and *energetics* (Kurth, 1931; see also Krebs, 1998). His approach revolves around the concepts of tension and release, energy of movement, and a feeling of supporting unity. From the depths of life energy, he states, various forms of expression of physical and mental forces emerge, with the energy of movement as the most remarkable and richest one. This embraces the basic feeling of action or work, which can be recognized as a sustaining force (Kurth, 1931, p. 77).

The analogy with physical forces is obvious here, especially if one conceives of psychic energies in terms of tension, movement, weight, buoyancy, pressure, etc. Both kinds of forces, however, are clearly not identical, although they can be seen as different manifestations of a common, unknown effect, which can either be experienced psychologically or perceived as forces in the external world (Kurth, 1931, pp. 98–99).

### 2.4. Phoronomy and the Genetic Definition of Geometric Figures

Central to Kurth’s approach are underlying tensions. They arise in an ongoing way and affect the flow of energy, but they also have the tendency to have a unified effect both in simultaneity and in succession, not as a subsequent connection but as a sustaining unity (Kurth, 1931, p. 32). This notion of unity, moreover, is of paramount importance, as it refers indirectly to the musical concept of form, which is based on an (unconscious) spatial concept. There are multiple technical terms and expressions, such as interval, distance, melodic range, parallel and counter movement, etc., that can be traced throughout music theory and demonstrate dark but deep-seated interweaving with spatial impressions (Kurth, 1931, p. 118). Such spatial impressions, however, only emerge when one goes beyond the individual notes to move on to concepts that are somehow connected with *movement* in music. The idea of space, e.g., is already implied in every melodic structure, as the term “melodic line” indicates quite clearly. But, as an expression borrowed from geometry, it has also its imperfections and shortcomings. Music, therefore, has its own geometry, which is different from the external spatial perception. It determines the structures of intervals, chords, melodic progressions, etc., and this gives rise to completely different laws for its internal dynamics and also for the symbolism of sound (Kurth, 1931, pp. 118, 121).

**Figure 6:**
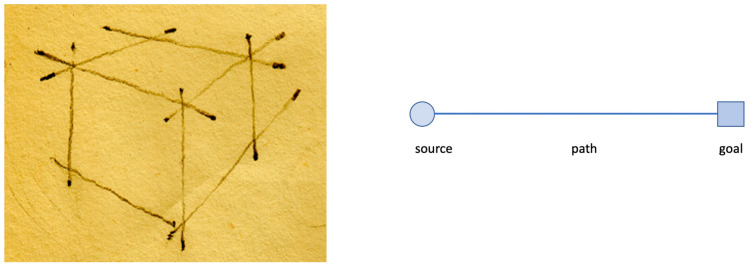
Example of a pencil drawing of a cube with clear marking of starting and ending points of the individual lines (**left**) as an illustration of the “source-path-goal” image schema (**right**). (Copyright © 2025 by the author).

Conceiving of music in terms of movement, furthermore, is an interesting starting point, especially when the movement is visualized by providing some lasting trace. This can be done in one, two, or more dimensions, and the most elementary way is the *drawing of a line*. Such a line, once drawn, is a static object, but it is also the outcome of the movement of its generation, and its shape can be modified by a variety of forces that are applied to it. This holds for one-dimensional lines but also for surfaces that are engendered by the movement of lines (Comte, 1830/1998, p. 162). A line, then, can be described in terms of a *path-function* that begins at a reference point and ends at another point that functions as a goal (Jackendoff, 1987, p. 153). It is one of the image schemata—*source–path–goal*—proposed by Johnson (Johnson, 2007, p. 144) that can be conceived as a structure for organizing our experience and comprehension. Such a schema captures the structural contours of our sensorimotor experience by integrating information from multiple sensory modalities and functioning as continuous and analogue patterns that operate beneath the level of conscious awareness. The schemata are “embodied,” which means that they arise from having a human body, with metaphorical projections from the kinesthetic source domain (Barsalou, 1999; Lakoff, 1993; Lakoff & Johnson, 1999). Figure 6 shows an example. It depicts the sketches of a figure drawing of a cube where lines are drawn by connecting two or more reference points that direct the movement of the drawing pencil. The result is a static picture, though it is possible to trace the lines again in one’s imagination so as to reconstruct the drawing by re-enacting their genesis, either through the real-time analogy of actually drawing or in a more compressed temporal format in imagery.

This dynamic aspect of the metaphorical projection of a line can be generalized more broadly to the generation of a curve, which can be regarded as engendered by the movement of a point subjected to some lawful constraints.

Grasping this actual tracing of a curve highlights the role of the embodied prehension of geometric figures as well as the forms of their surfaces, which are the product of the combination of certain elements of construction—e.g., points or lines—and movements. They echo what the ancient Greeks knew as the method of *genetic definition* (K. Lewin, 1952) and corresponds to the so-called “dynamic” vision of axiomatics in geometry (Defrise, n.d., p. 30).

**Figure 7:**
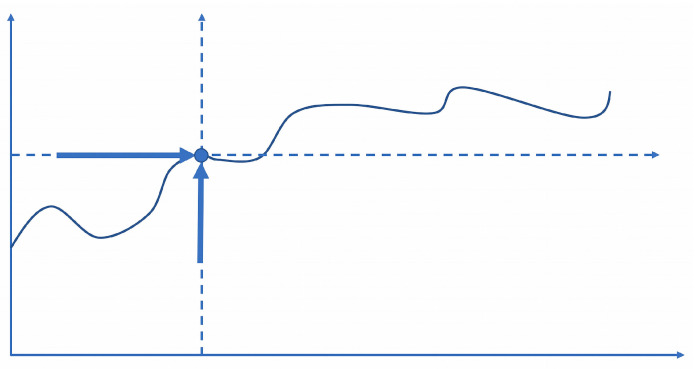
Phoronomic interpretation of a curve as the result of two perpendicular movements along the two axes of the figure. (Copyright © 2025 by the author).

The genetic construction of a curve has an obvious analogy in the statistical technique of plotting a curve. It relies on Descartes’ method of coordinates, which was a generalization from attempts to translate the form of a curve or the position of a point into numbers and words (D’Arcy Thompson, 1917/1942, p. 1032). The positioning of an object against a system of coordinates, however, can be conceived in a static or dynamic way. Figure 7 provides an example of the latter. It depicts the motion-related process of generating a curve, which can be described in *phoronomic* terms as having arisen from two mutually perpendicular movements of a point, which correspond to changes in the two variables of the curve (Colerus, 1935, p. 313). The term was introduced by Kant (Kant, 1786/1900 and Friedman, 2013 for a critical discussion) and is considered a philosophical and scientific concept that deals with the study of movement in a purely geometrical sense, excluding the influences of forces or other physical properties. The concept is not (yet) commonly used in music-related contributions, but the term is intuitively appealing, especially when trying to translate the felt experience of sounding music in terms of manifest or imagined movement, which brings us to the subject of sensorimotor integration and ideomotor simulation.

### 2.5. Sensorimotor Integration and Ideomotor Simulation

All of the above sounds quite abstract. The question, therefore, is how to translate this to the realm of music. There is, first, the possibility of “moving” and “being moved” by the music, either in an active or passive way. Music thus seems to have inductive power (Reybrouck & Eerola, 2017), and the evoked bodily reactions may motivate listeners to reflect on the sensations they experience. Listeners may literally feel sensations of being touched by the sounds, not only because of stimulation of the eardrum but also because their body senses kinesthetically the dynamic and temporal flow of the music (Meelberg, 2021). Such kinesthetic sensations can be explained by the functioning of the mirror neurons (see above) that are responsible for similar neural activity when actions are imagined instead of being performed in a manifest way. It means also that merely watching the movement of another can lead to sensing the movement as if one is performing the movement with one’s own body. It explains also the added value of attending a live performance compared to mere listening.

The act of performing movements at the virtual level in imagination has received some traction in the field of *ideomotor simulation* (Prinz & Chater, 2005; see Reybrouck, 2001 for musical applications). As opposed to mere imagery that functions as a perceptual sensation in the absence of corresponding sensory input, there is the possibility of imagery that is co-perceptual in the sense that it occurs simultaneously with sensory input. Imagery, then, may be helpful in structuring and organizing the actual experience over time by mediating between the perceptual input and the imaginative projections that go beyond the sensory processing of each actual moment in time. As such, it is to be distinguished from *sensorimotor activity*, which is “conservative” in the sense that it keeps step with the articulation over time. Sensorimotor strategies act mainly as a *controller* by linking the sensors to the central nervous system and its effectors. They work like cybernetic loops to keep possible deviations from the intended motor act within critical limits—hence the term “conservative”—and are working in a continuous and ongoing way, somewhat similar to servomechanisms that process sensory signals, which are transformed into motor commands through the estimation of errors and are regulated by feedback or feedforward mechanisms (Berthoz, 1996, p. 102).

It is tempting to relate these sensorimotor claims to the concept of *enactive listening*, which tries to “enact” the musical experience as if the listener should be performing him/herself (see Reybrouck, 2021 for a broad overview). Real enactive listening, however, is likely to be proactive rather than merely conservative (M. Jones, 1987, 2019; M. Jones & Boltz, 1989; Narmour, 1990), allowing the listener to anticipate the evolution of the sound on the basis of an internal model. It is a conception that fits in with newer paradigms in neuroscience that transcend the conception of the brain as a mere reactive machine with perceptual predispositions that act as selective filters and motor automatisms that are triggered in a quasi-automatic way (Paillard, 1994, 1990, 1977; Berthoz, 1997). This dispositional machinery has to be supplemented with a representational memory that forms the material for those mental operations that allow for cognitive control and anticipation. The human brain, in that view, is a *predictive machine* that controls direct reactivity to external solicitations by means of internal dialogues that are the hallmark of mental activities and symbolic play. There is, then, a transition from mere *sensorimotor integration* to *ideomotor simulation*. The former acts as wired-in programs that unfold in a quasi-automatic way, with a corresponding integration of sensorimotor dialogues with the self-organizing capacities of the nervous machinery (Paillard, 1994, p. 929); the latter involves a mode of functioning that predicts future states by relying on higher-level internal loops without direct links with sensors and by selecting reference frames for the control of movement and posture. It allows the brain to work not only as a *controller* but also as a *simulator* (Berthoz, 1996, p. 102).

Sensorimotor strategies and ideomotor simulation, furthermore, are not opposed to each other, but they can act in a complementary way to mutually reinforce each other. It seems, however, that sensorimotor strategies have received growing traction in current research by emphasizing how action can be perceptually guided in a perceiver-dependent world and how it can be subserved by being grounded in sensorimotor coupling and sensorimotor contingencies (Engel, 2010; Engel et al., 2013; J. O’Regan & Noë, 2001a; K. O’Regan & Noë, 2001b; Cummins, 2013; Cummins-Sebree et al., 2007). Hence, also, the growing role of *perception and action* studies (Prinz & Bridgeman, 1996). The ideomotor simulation that relies on internal models, on the contrary, has been criticized to some extent, especially from the field of radical embodied cognition (Baber, 2021; Baggs & Chemero, 2021; Chemero, 2009), which holds a position that argues against doctrinal internalism by endorsing an alternative view of motor action that downplays the role of conceptual activity and representation in favor of a dynamicist and enactive approach that relies on closed-loop coupling with the environment through sensorimotor interactions. The position is radical in the sense that it argues for a quasi-exclusive replacement of representationalist or computationalist positions by dynamic and interactional methods of coping with the environmental world (Hutto & Myin, 2013).

Music processing—either as performer or as listener—is an interesting case in this regard. It relies on both sensorimotor and ideomotor activity, the former as a conservative process that keeps step with the sonorous articulation in an attempt to stay as close to it as possible and the latter as a predictive process that allows for predictions about the future unfolding of the music. The combination of both strategies makes it possible to process music in a perceptual and imaginary way, thus assaying both the subtleties of the sonorous articulation and the more abstract internal dialogues that allow for simulation of how the music will go.

There is, furthermore, a less explicit level of ideomotor simulation, as advocated in the above-mentioned “energetic” approaches to music psychology. Kurth and Mersmann, among others, have stated that there is a more general experience of ideomotor simulation contained in the listening process that can be experienced as “forces” and “energies.” These are exemplified most typically in those musical structures that may account for our perception and imagination of “tension,” “dissolution,” and “movement” (Kurth, 1931; Mersmann, 1926). This is an interesting conceptual move, as it opens up the elusive problem of the distinction between “inner” and “outer” movements, as initiated by Becking, Sievers, and Truslit, who pointed to the idea of capturing the rhythmic content of tone and word poems through accompanying movements (Becking, 1928; Sievers, 1924; Truslit, 1938). Sievers, in particular, was guided by an interest in sound analysis with the aim to uncover previously unknown properties of the sonorous mass through observation and experimentation.

## 3. Dynamics of Listening

Music, as a temporal art, presents itself under a double guise of the temporality of the music itself—allowing us to compare it with an organism—and ongoing processing by the listener. This temporality is its defining feature, leaving pitch, duration, loudness, and timbral characteristics only of secondary importance. Sound, moreover, is the medium or carrier through which the temporal events are organized, but it is by itself an insufficient definition of music. Its principal characteristic, according to Serafine, is movement in time, which she defines as “the exploration of simultaneous and successive events that embody points of arrival and stasis, points of departure and continuation, and a train of event-to-event similarities and transformations” (Serafine, 1988, p. 69). Music, then, can be seen as an organic process with a temporal signature that can be translated rather easily in movement patterns, which may be both virtual and manifest.

### 3.1. Music as a Continuous Function of Time

A major challenge in defining the temporality of music is the tension between *potentiality* and *actuality*, which, according to Whitehead, has permeated some chief notions of European thought for centuries. Continuity is concerned with what is potential, while actuality is incurably atomic. There is, as such, a perceptive mode with clear consciousness of the extensive relations in the world, which includes the extensiveness of space and time. Such clarity is obtained in ordinary perception through the senses and termed, therefore, *presentational immediacy*. This means that the contemporary world is consciously prehended as a continuum of extensive relations (Whitehead, 1929/1957, p. 77).

Another philosophical challenge is Bergson’s charge that the human intellect “spatializes” the universe by ignoring its fluency and analyzing the world in terms of static categories. Western philosophy had to wait for Newton, who brought fluency back into the world of absolute mathematical time with a mathematical description in his *Theory of Fluxions* (Whitehead, 1929/1957, p. 242).

There is no space to go into details here. The claim, however, is of considerable relevance, and it can be asked whether music can be conceived as a *(continuous) function of time* and whether there is an expression of *organic life* in the accomplishment of music. The analogy with a mathematical curve is obvious, where every movement of a point can be conceived as if it followed a more or less complicated law with a corresponding arithmetic expression in the conditional equation or function.

The analogy may seem trivial, yet it is fruitful in its simplicity. The question can be raised, however, as to whether music—as movement in time—can be represented as a two-dimensional curve. Considering also the materiality of sound, it seems arguable to conceive of it rather in terms of a three-dimensional representation in a kind of virtual space. There is, moreover, the danger of a rather naïve geometric representation that lacks an in-depth mathematical and dynamic justification.

Motion representation in a visual display, furthermore, is selected by convenience, which means that it needs to distinguish only between phenomena of interest. These may incorporate rudimentary notions of direction, velocity, and shape (Badler, 1986). Perceivers, moreover, identify a moving object as seen against a changing background. This results in a coherent flow of that object, with the common motion of its elements being displayed by the vector path that is shared by all elements. The relative motions of the elements are then seen as residuals that move with respect to the whole. There are also two types of displacements, namely, those relative to the object and those relative to the observer (Cutting, 1986).

This brings us to the question of what in music are the real *bearers of form*. Some answers were given already in the 1980s. Shepard, among others, has stated that among the potentially variable attributes of tones, such as loudness, timbre, and perceived spatial location of source, pitch and time play a most essential role. They appear to be unique in the auditory modality as bearers of form, or, to adopt Attneave’s term, they are *morphophoric*. This means that certain psychophysical continua are the media in which patterns occur, and the appropriate scale for any such medium is one that reflects the invariances preserved in transposition behavior, where the relationship between stimuli remains unchanged, even when the absolute values of those stimuli change (Attneave, 1972; Attneave & Olson, 1971; Shepard, 1981, 1982a, 1982b).

Much more can be said about these bearers of form. Truslit, again, was rather critical about giving such a prominent role to pitch. Timbre and pitch, he argues, are part of the musical design, but their importance is subordinate to that of *dynamics* and *agogics*. This is exemplified in the failure to make a piece of music alive only “according to the notes” as depicted in a score but without the dynamo-agogic subtleties and relying only on the changes of pitch or timbre. Dynamics—the subtle changes in loudness, such as increasing/decreasing—and agogics—the subtle changes in tempo, such as acceleration/deceleration—work as a unity to guide the movement and give it its power or energy. As acoustic means of musical design, they manage the dynamic development over time and function as the expression of an inner experience expressed in movement (Truslit, 1938, p. 31 and 60–63).

The analogy is challenging. It raises the question of how to translate the depiction of the music as an imaginary curve into the gestural approach of physical motion, either imaginary or manifest. It is an approach that can even be connected to more recent work that engages with contemporary computational models that contrast their phenomenological approach with formalist ones (see Mazzola & Andreatta, 2007 for an in-depth discussion).

What is typical of gestures, moreover, is their continuous design. As such, they align with natural processes, which, as a rule, proceed in a “continuous” way. Our ordering mind, on the contrary, thinks more readily in terms of “discrete” categories, in the sense that our understanding has established a dividing principle by using number and quantities, where the reality of observation meets and needs continuity (Colerus, 1935, p. 298). In nature, everything moves in curves—a planet that follows its orbit, a fired bullet, a hurled boomerang, etc.—but what does it mean to move in curves that should be considered expressions of musical movement? Its main purpose should be to activate the body and to let it merge with the musical movement so as to experience the music not only with the ear but also with the body as a whole (Truslit, 1938, pp. 107–109).

It is thus possible to represent the movement of a melodic sequence not merely through the differences of pitches but by grasping its overall movement, which can then be expressed by a continuous curve. This means that movement is performed in such a way that the path described by the moving body generates curves, which are composed of parts of a circle or an ellipse so that the transitions between these parts occur smoothly and imperceptibly (Truslit, 1938, p. 78).

**Figure 8:**
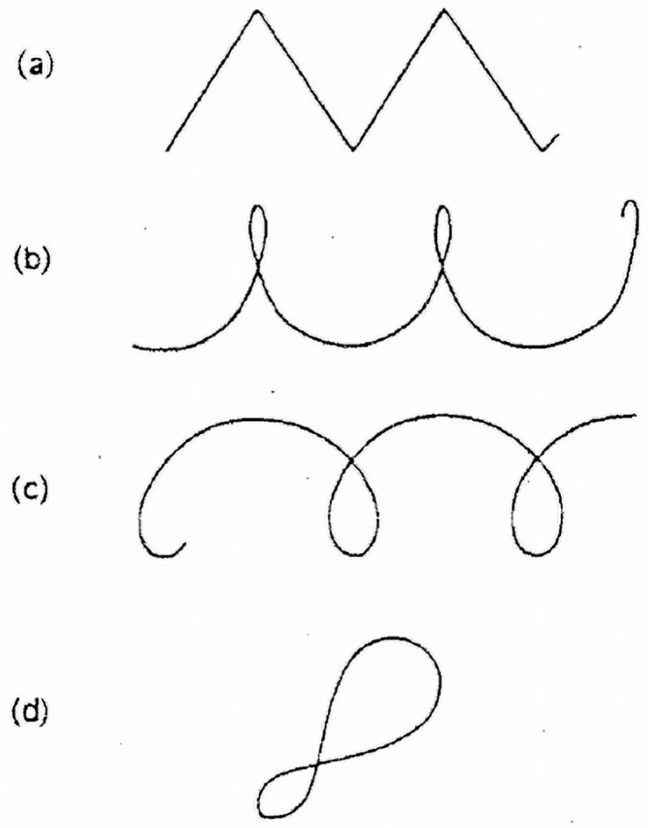
Depiction of Truslit’s basic movement curves: (**a**) straight, (**b**) open, (**c**) closed, and (**d**) winding. The first curve is artificial, and the other ones are natural (Freely adapted from Truslit, 1938, Copyright © 2025 by the author).

Truslit has delved deeply in the drawing of curves when listening to music, arguing that it is in the nature of our functional organization that the general direction of movement must agree with the direction of the sequence of tones, aligning the rising and falling of the pitches with the rising and falling of the movements. He thus distinguished some basic possibilities for suitable curvilinear trajectories that correspond to the natural forms for an upside–down movement, which he termed, respectively, straight, open, closed, and winding movements (see Figure 8).

The open movement begins calmly, accelerates while ascending, makes a narrow counter-clockwise loop, and decelerates on the way down; the closed movement begins rapidly, decelerates as it reaches the top, and accelerates on its way down, making a larger clockwise loop if it continues into another movement; the winding movement ascends diagonally into a large counter-clockwise loop and descends almost vertically, making a smaller clockwise loop at the bottom, which leads it back to its origin (Truslit, 1938, p. 79).

The curvatures have innumerable variants and can be combined in multiple ways. It should be mentioned, however, that the design of a beautifully curved line is not important for its own sake. It should be seen only as a model for the movement sequence and as a means for communication and visualization. What really matters is the experience of the natural movement of the music, and the curved image must be understood as a process or trace of something that is moving and experienced as its image (Truslit, 1938, p. 110).

**Figure 9:**
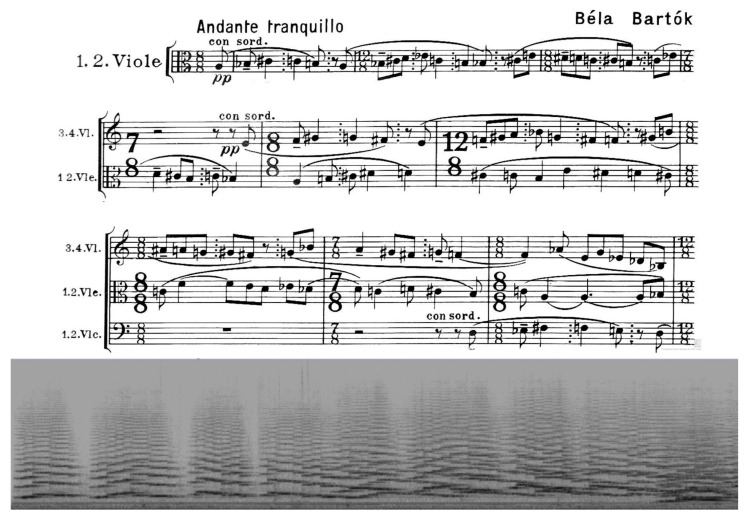
Example of visualization of the first 9 bars of the first movement of Béla Bartók’s *Music for Strings*, *Percussion and Celesta*. The upper pane shows the score notation, and the lower pane shows the spectrogram. (Copyright © 2025, IMSLP-Public Domain and by the author).

All of this may seem to be quite speculative. It is interesting, therefore, to compare Truslit’s intuitions with existing visualizations of music, both in a discrete and continuous way. Figure 9 provides an example. It shows again the first bars of the first movement of Béla Bartók’s *Music for Strings*, *Percussion and Celesta* (see also Figure 2). The fragment has a duration of about 47 s and is depicted as both a score notation and a spectrogram. The score notation is an obvious example of the discrete approach, with each note being depicted as a separate and distinguishable entity. The spectrogram, on the contrary, shows a more continuous image. There is no clear distinction between the notes, and even at the division points between the broader arches, there is hardly any blank space on the spectrogram. It is possible, however, to discretize this continuous depiction in our mind by imposing a kind of temporal grid that corresponds to the temporal resolution of the note values. The most natural way of listening, however, is to follow, as it were, the curvatures of the fundamentals and the overtones, which show a clear visual curvature. It is striking, moreover, that there are also phrasing arcs on the score notation that match pretty well with the curvatures on the spectrogram. It is not difficult, furthermore, to imagine ways of externalizing this curvature-based way of listening or watching through overt motor behavior. The art of conducting can be taken as an example here, though conducting is much more than merely mimicking melodic curves.

### 3.2. Sound Tracking and the Consumption of Time

The experience of music as something that moves through time has epistemological implications. This means that its constituting elements only exist because our consciousness passes through them, and this “passing through,” or *discursus*—to use Cassirer’s term—defines the concept of time itself as a “being of succession” (Cassirer, 1922/1977, p. 170). It is a methodological approach that goes beyond a conception of consciousness as a fixed and rigid state and of the processes of consciousness as a mere sum or combination of states, which is the hallmark of sensualistic psychology. Starting from movement and the feeling of that movement, on the contrary, is a fundamental factor in the structure of consciousness. This means that the dynamics of unfolding are not based on statics but that all reality of our psychic life consists of processes and changes (Cassirer, 1922/1977, p. 127).

Listening to music, accordingly, cannot be reduced to instantaneous perception. It needs, on the contrary, some accumulation over time and a tendency to saturation, which is related to good continuation, completion, and the fulfillment of expectations. There is, as such, a normal expectation of progressive change and growth (Meyer, 1956/1961, p. 135), as well as the special nature of the typical relationships of time. Contrary to relations of space, where the “here” and “there” have only a simple distance relationship between two separable spatial points without any preferred direction and with the possibility of being perceived together, time shows not only the separation and mutual distance of its individual elements, as it also has a unique and irreversible meaning, with the direction from past to future or from future to past being unmistakably its own (Cassirer, 1922/1977, p. 173).

This special status of the experience of time is also expressed in the grammar of language, with the tenses of verbs as an example of categories that express a reference to time. There is, as such, the distinction between the perfective and imperfective, the momentary or cursive, the one-time or iterative type of action. An important difference is whether the action is complete and finished at the moment of speaking or whether it is still in development, whether it is limited to a specific moment or extended over a longer period of time, and whether it is carried out in a single or repeated act (Cassirer, 1922/1977, p. 216). Action verbs, or dynamic verbs, describe the action that one is performing, in contrast to stative verbs that describe a state of being or perception. They can refer both to physical and mental actions, such as internal processes and actions that are related to thinking, perceiving, or feeling, but they can be described also in terms of spatial positions, with a division into three important classes, namely, GO verbs, BE verbs, and STAY verbs (Jackendoff, 1987, p. 153).

It is not difficult to imagine music as consisting of sounds that “go” from here to there. Sounds that are logically discrete and isolable can then be perceived as a continuous gesture in a continuous sweep so that what comes earlier is tied to what comes later, even if the former has stopped sounding already (Serafine, 1988, p. 75).

A fruitful analogy to make this more concrete is the concept of *sensory tracking*, as studied in the visual domain. Visual tracking simply means that a person keeps an object that moves in the visual field in focus by moving their head or at least their eyes. We could define sound tracking accordingly as the process of directing our attention in a continuous and ongoing way to the real-time unfolding of the music through time. It is an important aspect of the structuring of time.

This process of sound tracking, as we see it, is processual, ongoing, and continuous. It proceeds in real time, and it is a consummatory experience in a Deweyan sense, not subordinated to operational thinking but as an end in itself and with an element of fulfilment (Dewey, 1934/1958; see also Määttänen, 2000). It is a typical example of listening to music in terms of action verbs, with the tracking process being seen as the externalization of a felt experience, both to ourselves and to others.

This holds for listeners but also for performers, who are also always listeners. It is exemplified quite typically in Dhrupad music pedagogy, which, as one of the oldest vocal styles of Hindustani classical music, is characterized by smooth, slow, melodic glides, whereby pitch is conceived as a continuum and melody is conceptualized as movement within an imaginary “pitch space” (Fatone et al., 2011). Vocalists seem to navigate this imagined pitch space through hand movements in the real three-dimensional space, with observable relationships between hand movements and melodic patterns (Paschalidou, 2022; Clayton et al., 2005). This connection even goes beyond mere spatial Euclidean geometry in the sense that it extends to the sensation of forces and effort. Singers, therefore, report that they frequently engage with melodic patterns as if they are manually sculpting spaces through manual interactions with imaginary objects that are physically tangible. These objects can be imagined as an elastic band, a ball, water, etc., but, as a rule, they pose a distinct sense of resistance and afford various interactions, such as stretching, expansion, compression, pulling, throwing, and others. Motor imagery, in that case, is then “materialized” through effortful physical actions directed towards them.

## 4. Conclusions and Perspectives

In this paper, we have described listening in rheological and action terms. It is an approach that fits well with current theoretical frameworks of embodied and enactive cognition that have revolutionized somewhat the last decades of cognitive science. These so-called “new” paradigms, however, are not quite so radical and innovative as commonly thought. Careful investigation of the extant literature shows remarkable parallels with earlier contributions in the 1930s and later, which are often cited rather than being read. Going through these seminal works, however, is quite refreshing and even illuminating in the sense that many of these early scholars had ideas that were appealing due to their breadth of scope and because they were not hindered by the methodological constraints of current scientific research and therefore often unprecedently groundbreaking. Their lack of scientific rigor has often been used as a reason to dismiss them as unscientific and thus also as irrelevant, with many fruitful and inspiring ideas having been left unexplored.

Reading these original sources, therefore, is enlightening in the sense that many of these ideas are not only original but also quite modern, sometimes anticipating current insights by decades. Many of them have passed through our review: Truslit, Becking, and Sievers, who elaborated in depth on the gestural approach to music as movement in time; Cassirer, for his elegant and intuitive comments on the mathematical concept of function and the dynamics of processes and changes; Dewey, for comments on the river metaphor and the distinction between flux and continuity; Kurth and Hartmann, for their emphasis on the dynamics and energetics of mental processes; D’Arcy Thompson, for his studies on growth and form; and Goethe, Michaelis, Coleridge, and Assafjew for elaborating on the concept of organic form, to mention some of them.

Our overview, therefore, can be seen as an attempt to revitalize some of these earlier claims with the aim to energize current foundational work on musical experience by introducing some tried and tested descriptive and explanatory frameworks. However different these may be, they all converge around the temporal character of music in its sonorous unfolding, thus describing musical narrativity from an experiential point of view, with the aim of exploring what music “does” or “can do” to the listener, rather than providing an analytical description of what it “is” (Meelberg, 2021).

Music listening can then be understood in analogous terms as an act of reconstructive doing where “real” perception, as Truslit conceived it, implies an act of listening to arouse vivid consciousness and a lively, felt experience. This might involve the cooperation of motor elements, even if these may remain implicit or do not become manifest. It is a conception that echoes Truslit’s claim that true musicality is the ability to experience and create music through movement, not understood in terms of the technical skill for mastering a musical instrument or other performance skills but as being constitutive of “real” musicality (Truslit, 1938, p. 201). It suggests that any movement can be expressed acoustically through dynamics and agogics, thus reducing somewhat the meaning of every musical design to its movement design (Truslit, 1938, pp. 59–60).

There is, finally, also the tension between the inner and outer experience of a felt experience. Most of the earlier scholars emphasized the major role of grasping the musical form that is formed “from within” as the form that is essential to the whole and that points beyond itself. Such an inner form is to be contrasted with a mere accidental external form. It is supposed to constitute the formative principle of the external and should be considered one of the core ideas of the aesthetics by Hartmann (Hartmann, 1953). Or, as Kurth puts it, the actual bridge to the inside is built by the sensation that reacts to the external stimulus. It is a conversion that highlights the psychological part of perceiving and that shows the importance of building bridges between inside and outside. It is exemplified in the close connection between the spheres of physical and psychic forces, which can be found in the movements of the human body itself, including many unconscious muscle sensations. They are reflected in terms like tension, relaxation, and movement, suggesting that physical movements are also triggers of psychic impulses, though it would be false to imagine the basic psychic movements simply in externalization through physical movements (Kurth, 1931, p. 100).

This brings us to an aspect that was not really covered in this paper: how to relate emotional and motor tensions in music. It is a question that explores the animate character of the materiality of sound and that fits in smoothly with current empirical research on the inductive power of music (see Reybrouck & Eerola, 2017 for an overview) in an attempt to revitalize the definition of music as the unmediated language of affects (Michaelis, 1801).

It thus seems that there are still plenty of things to investigate. Most of them can be formulated as pending hypotheses that are waiting for additional empirical research. Some of them have been formulated already in intuitive terms or by using metaphoric language, but most of them still need more substantial grounding. The list below provides a non-exhaustive list of questions and/or statements that can be seen as perspectives for future empirical research:

- Can all music be conceived as flowing energy? What about percussive sounds and music with an abundance of beats? Is it possible to generalize from music that typically flows organically to other kinds of music?
- What is the relationship between rheological and phenomenological listening?
- How discrete is discrete? Zooming in on the sonorous unfolding shows a transition from a point in time to an event with some temporal unfolding (Δt). The momentary then takes on a rheological signature.
- Is it possible to refine the process of listening by learning to listen as if we listen in slow motion to the actual unfolding? Is this the hallmark of expert listeners and/or top performers? Is this related to the time frame of perception, and is it possible to modify this frame through training and attentional focus? (see for example, Rammsayer & Altenmüller, 2006; Kraus & Chandrasekaran, 2010).
- High-resolution listening is rheological listening. It increases the number of perceived sounding elements to such an extent that the discrete elements transform into a continuous flux.
- How can we translate the flow of sounding events in rheological terms? How can we provide a quantitative description of the sounding flux? Potential parameters are speed, volume, flow, acceleration, resistance, viscosity, elasticity, etc. How can we translate these physical parameters to the realm of music without losing the rigor of scientific definitions?
- Can the river metaphor be operationalized in terms of morphodynamics or hydrodynamics as applied to music as a sounding flux?
- The existing research on beat induction and entrainment should be broadened to encompass also non-rhythmic and non-metric music, even including natural and artificial sounds.
- What is the impact of defining music as vibration in the vestibular response? Does vestibular activation also occur at low levels of sound intensity? Can we conceive of vestibular responses in cases of mere listening without manifest motor behavior?
- How to refine the measurement of the vestibular responses, both in a direct and indirect way. What are future perspectives on using cochlear and vestibular myogenic responses?
- To what extent is the vestibular response able to modulate the sensory input and alter the aesthetic response by the listener? What is the functional significance of the efferent vestibular system compared to the cochlear responses of the inner ear?

The overarching picture that emerges at the moment is that there are actually more questions than solutions. It seems plausible at the current stage of investigation that bodily movement to music—especially active movement—can influence the perception of the music, but the question can also be reversed: does music listening evoke motor responses, either manifest or virtual? And in the case of the latter, does this “as-if” character of motor simulation involve the engagement of the vestibular system and its possible relations with proprioception and kinesthetics? It has been shown that the perception of rhythm in infancy involves interactions between the developing auditory, motor, and vestibular systems, with the initial sensorimotor system of the infant being weakly and diffusely coupled at that developmental stage (Cirelli & Trehub, 2019; Phillips-Silver & Trainor, 2005; Sheya & Smith, 2010; Tichko et al., 2021). It has also been hypothesized that connections between auditory and motor networks are not unidirectional—activity from the motor network shaping activity in the auditory network—but bidirectional (Gerry et al., 2010).

There are currently some significant developments in this regard. Vestibular research has seen a significant backlog of cochlear research, however, due to the difficulty of recording vestibular activations. Where cochlear activation can be routinely recorded via a round window electrode—the round window is one of the two openings from the middle ear into the inner ear—vestibular activity requires much more complex surgical exposure of the vestibular end organ. Vestibular research, therefore, has very few measures available to examine the electrophysiology of the peripheral vestibular function in living humans or animals. Researchers initially relied on single neuron recordings or recordings of the Vestibular short-latency Evoked Potential (VsEP) (T. Jones, 1992; T. Jones & Jones, 1999), but several new experimental measures have been developed more recently (see Curthoys et al., 2021 for a broad overview). Among these are recordings from the utricular macula, including the Utricular Microphonic (UM), the Utricular Summating Potential (Pastras et al., 2017, 2021), and the Vestibular Neurophonic (Ashcroft & Hallpike, 1934), all of which provide first-order, physiological assessments of utricular function. They also have clinical value, as they are related to the vestibular evoked myogenic potential (VEMP), which is a clinical tool for testing the evoked otolithic response to (abrupt) sound and vibration by measuring the myogenic potential recorded from tensed muscles, such as the sternocleidomastoid neck muscles (cervical VEMP or cVEMP) and the inferior oblique eye muscles (ocular VEMP or oVEMP) (Curthoys et al., 2021; Colebatch et al., 1994; Curthoys et al., 2021; Rosengren & Colebatch, 2018). The VEMP can be considered the clinical analogue of the Auditory Brainstem Response (ABR) for testing the otolithic response to sound and vibration. One must keep in mind, however, that much more vibratory energy is required to evoke a vestibular response compared to an auditory evoked response.

Summarizing a little, it can be stated that not all hearing is cochlear. This was a major claim by Truslit (1938) and Tait (1932) that was picked up again in the 1960s and 1970s in research on vestibular acoustic sensitivity. It culminated in the discovery of both the inian response—electrical signals detected in the area around the inion, a protrusion on the back of the skull—which was found to be vestibular and myogenic in origin (Bickford et al., 1964; Townsend & Cody, 1971) and the vestibular evoked myogenic potential (VEMP), which is a vestibular reflex that is mediated by the acoustic sensitivity of the otolith organs and the vestibular spinal tract (Colebatch et al., 1994). These measurable potentials are important electrophysiological tools to address the question how otholitic receptor cells are activated by both sound and vibration. The VEMP, furthermore, has been developed as a non-invasive clinical tool and also as a scientific tool to investigate acoustic sensitivity of the otoliths to both air- and bone-conducted sound (Todd & Lee, 2015).

All of these findings raise questions that clearly show that there is still a long way to go, but they also show that parts of the answers have been anticipated at least conceptually in many earlier writings. We hope that the findings that we brought together in this paper may open new avenues for future theoretical and empirical research.

## Data Availability

Not applicable.
